# Hydrogen peroxide signal photosynthetic acclimation of *Solanum lycopersicum* L. cv Micro-Tom under water deficit

**DOI:** 10.1038/s41598-023-40388-y

**Published:** 2023-08-11

**Authors:** Gustavo Ribeiro Barzotto, Caroline Pardine Cardoso, Letícia Galhardo Jorge, Felipe Girotto Campos, Carmen Sílvia Fernandes Boaro

**Affiliations:** 1 Plant Production Department, School of Agriculture, UNESP—São Paulo State University, Campus Botucatu, Ave. Universitária, n° 3780‐Altos do Paraíso, Botucatu, São Paulo 18610‐034 Brazil; 2 Biodiversity and Biostatistics Department, Institute of Biosciences, UNESP—São Paulo State University, Campus Botucatu, Street Prof. Dr. Antonio Celso Wagner Zanin, 250‐District de Rubião Junior, Botucatu, São Paulo 18618‐689 Brazil

**Keywords:** C3 photosynthesis, Drought, Plant physiology

## Abstract

The current climate change setting necessitates the development of methods to mitigate the effects of water scarcity to ensure the sustainability of agricultural activities.f Hydrogen peroxide (H_2_O_2_) is a plant signaling molecule that can trigger metabolic defense mechanisms in response to adverse environmental circumstances like as drought. The purpose of this study was to investigate if foliar application of H_2_O_2_ stimulates modifications in photosynthetic metabolism for adaptation of tomato plants to a period of water deficit and recovery. The study, which was carried out in a factorial scheme, tested plants subjected to two water conditions (well-watered plants and plants subjected to water deficit), as well as foliar application of 1 mM H_2_O_2_ (zero, one, or two applications, 24 h after the first), and was evaluated in two moments, during the deficit period and after recovery. Foliar application of 1 mM H_2_O_2_ resulted in a 69% increase in the maximum rate of RuBisCO carboxylation in well-watered plants, contributing to tomato photosynthetic adjustment. H_2_O_2_ treatment resulted in a 37% increase in dry mass in these plants. In plants subjected to water deficiency, 2× H_2_O_2_ increased stress tolerance by reducing the maximal rate of RuBisCO carboxylation by only 18%, but in plants that did not receive H_2_O_2_ treatment, the reduction was 86% in comparison to the wet plants. Plants exposed to a water shortage and given 2× H_2_O_2_ stored sucrose in the leaves and had a 17% higher relative water content than plants not given H_2_O_2_. Thus, H_2_O_2_ foliar treatment can be used in tomato management to induce drought tolerance or to boost photosynthetic activity and dry mass formation in well-watered plants.

## Introduction

Water scarcity is one of the primary abiotic stressors that cause agricultural losses^1^, and in the present context of climate change, methods to mitigate the impact of water scarcity are critical for the long-term viability of agricultural operations.

Tomato (*Solanum lycopersicum*) is one of the most farmed vegetables in the world, with a harvested area topping 5 million hectares in 2021^2^. Although the expansion of tomato cultivation in a protected environment provides the opportunity to manage environmental conditions, most production occurs in the field, where the crop is mostly dependent on rainwater supply.

Drought stress in plants has been thoroughly documented in the literature, causing physiological, morphological, and biochemical alterations as well as decreased plant development. The oxidative damage generated by reactive oxygen species (ROS) and a rise in ethylene concentration, known as the stress hormone, can result in increased cellular respiration and chlorophyll degradation, among other consequences^3^.

Under water stress, the energy acquired in plant photosystems exceeds the capability for organic synthesis, as the reduction in CO_2_ supply due to stomatal closure inhibits photosynthesis. Under these conditions, the formation of ROS in chloroplasts increases^4^, as does the waste of energy for photoprotection, whether in the form of fluorescence, heat, or another electron drains such as photorespiration^5–7^. Other metabolic alterations include an increase in auxiliary foliar pigments, which serve to dissipate excess energy and eliminate ROS, and an accumulation of soluble carbohydrates, which act as osmoregulators. ROS generation rises as the stress period progresses due to decreases in photosynthetic activity and lipid peroxidation^4^.

Although excessive ROS generation in plants under stress causes damage that leads to decreased development, these molecules are required for stress signaling and identification. In chloroplasts, ROS generation ensures the creation of a large network of connections that allows the chloroplast's redox characteristics to operate and balance. Furthermore, numerous proteins are triggered by oxidative activities, forming an efficient type of signaling, particularly for regulatory mechanisms in CO_2_ absorption^8^.

ROS signaling does not occur exclusively at the chloroplast level, and the signal must be transferred from cell to cell as well as tissue to tissue in the plant body to have a systemic effect. Because it is more stable, H_2_O_2_ is the most active ROS in signaling pathways. Gilroy et al.^9^ and Campos et al.^10^ discovered a link between H_2_O_2_ generation and Ca^2+^ content in cells. The change in Ca^2+^ concentration works on kinases that create RBOH proteins (NADPH oxidase), which produce H_2_O_2_, increasing the concentration of this ROS in a manner that enhances signal maintenance. These events are communicated from cell to cell, resulting in a change in membrane potential and the formation of a wave that may propagate swiftly and efficiently. This system is critical for stress identification and fast signaling in response to changing environmental conditions^9^.

The involvement of H_2_O_2_ in plant signaling to abiotic stress suggests that it could be used exogenously to cause shifts in metabolism and aid acclimation^9,11,12^, which refers to defense preparation, with improved response capacity to the stress after initial signaling^13^. H_2_O_2_ causes the oxidation of cysteine residues, a recognized molecular switch implicated in various signaling pathways, resulting in the creation of disulfides mixed with glutathione, which are thought to shield proteins from further oxidation and modulate proteins that interact with DNA. Gene expression control enables rapid response without the requirement to manufacture new messenger RNAs and export them from the nucleus^14^. This signaling pathway has been characterized as crucial to plant innate immune system responses, and its key mediator is NADPH oxidase, which controls H_2_O_2_ generation and is required to modify defensive response intensity^15^.

H_2_O_2_ foliar delivery in drought-stressed plants has already been shown to be advantageous in other crops such as soybeans^16^ and cucumber^17^. Plants cultivated under normal water conditions, on the other hand, can benefit from H_2_O_2_ treatment by stimulating growth, as indicated by Jamaludin et al.^18^ For diverse plant species, H_2_O_2_ concentrations ranging from 0.05 µM to 200 mM have been recorded for adaptation to stress by environmental factors^14^. ROS and ethylene were also discovered to be important in enhancing tomato tolerance to salt stress caused by brassinosteroids (BR)^19^.

Thus, we propose that foliar application of H_2_O_2_ at a sufficient concentration might trigger metabolic changes in tomato plants cultivated under water shortage or regular watering regimes, resulting in reduced water lack damage and increased growth.

The aim of this study was to investigate if foliar H_2_O_2_ treatment increases photosynthetic metabolism adjustment for tomato plants subjected or not to a period of water shortage and recovery.

## Results

H_2_O_2_ foliar treatment enhanced the *V*_*cmax*_ of plants independent of water circumstances and maintained the *J* and *TPU* of water-stressed plants at levels comparable to those of well-watered plants.

**Figure 1:**
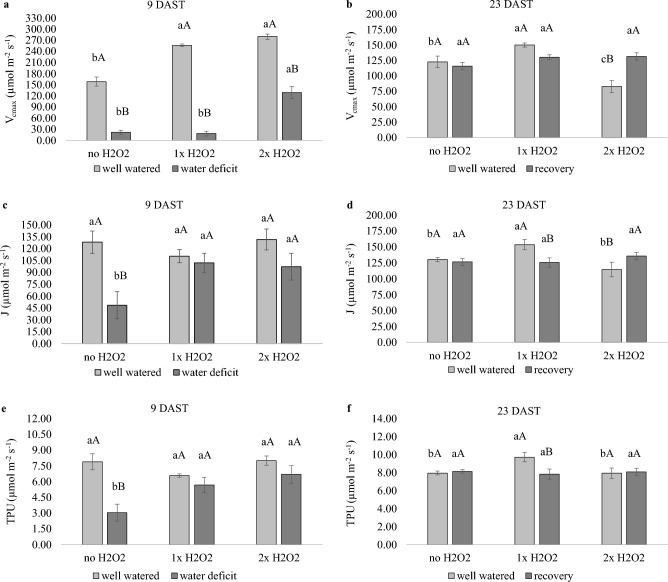
Maximum RuBisCO carboxylation rate (*V*_*cmax*_), RubP regeneration rate linked to electron transport (*J*), RubP regeneration rate linked to the use of triose phosphate (*TPU*) of tomato plants well-watered or subjected to water deficit (9 days after the treatments beginning–DAST)/recovered (23 DAST), and zero, one or two foliar applications of H_2_O_2_. Values correspond to the mean ± standard error (n = 4). Means with equal letters do not differ from each other by the Tukey test (< 0.05), uppercase between water conditions and lowercase between foliar application of H_2_O_2_.

Well-watered plants that received H_2_O_2_ had a greater *V*_*cmax*_ at 9 DAST (Fig. 1a), with an increase of about 69% compared to plants that did not get H_2_O_2_, and only 1× H_2_O_2_ plants had a higher *V*_*cmax*_ at 23 DAST (Fig. 1b). In plants with a water shortage, 2× H_2_O_2_ raised *V*_*cmax*_ by roughly 6 times relative to plants that did not receive H_2_O_2_ (Fig. 1a).

H_2_O_2_ administration to well-watered plants had no effect on *J* and *TPU* at 9 DAST (Fig. 1c,e), whereas plants that got 1× H_2_O_2_ at 23 DAST had increased *J* and *TPU* (Fig. 1d,f). by 9 DAST, the application of H_2_O_2_ quadrupled *J* and *TPU* values in plants subjected to water deficiency, but no effect was detected by 23 DAST.

H_2_O_2_ foliar application boosted the growth of well-watered plants while paralyzing the growth of plants subjected to water deficiency that received 2× H_2_O_2_, even though these plants had a higher RWC.

**Figure 2:**
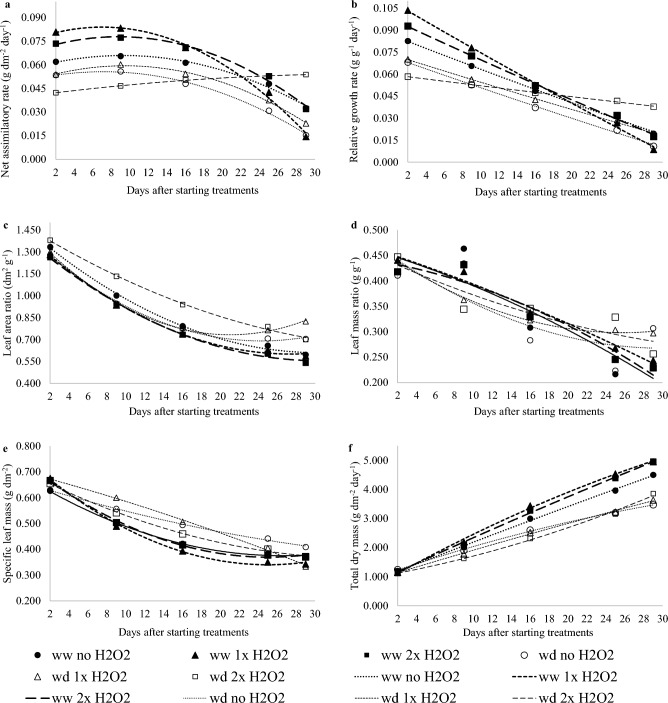
Growth rates of tomato plants well-watered or water deficient/recovered, and zero, one or two foliar applications of H_2_O_2_. The return of watering occurred at the end of the twelfth day.

**Figure 3:**
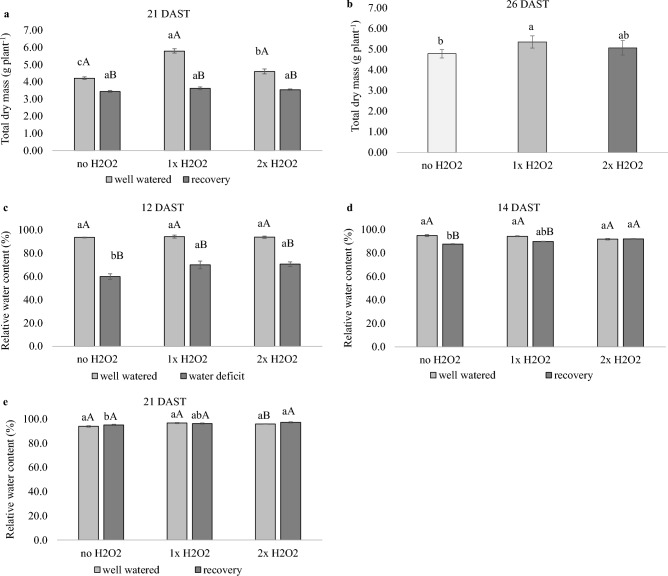
Total dry mass and relative water content of leaves of tomato plants well-watered or subjected to water deficit (12 days after the treatments beginning – DAST)/recovered (14, 21 and 26 DAST) and zero, one or two foliar applications of H_2_O_2_. Values correspond to the mean ± standard error (n = 4). Means with equal letters do not differ from each other by the Tukey test (< 0.05), uppercase between water conditions and lowercase between foliar application of H_2_O_2_.

Foliar H_2_O_2_ application in tomato promoted changes in plant growth, particularly in well-watered plants that received 1× H_2_O_2_, which grew faster, as evidenced by an earlier drop-in net assimilation rate (Fig. 2a) and higher dry mass (37% higher compared to plants that did not receive H_2_O_2_ application) (Fig. 2f and 3a,b), and in water-stressed plants that received 2× H_2_O_2_, which grew slowly (Fig. 2a,b). The net assimilation rate and practically constant relative growth rate over the time analyzed had the opposite result as the other treatments. Photoassimilates were maintained in the leaves of plants subjected to water deficit and treated with 2× H_2_O_2_, as demonstrated by a higher leaf area ratio, leaf mass ratio, and leaf specific weight (Fig. 2c,d,e).

Plants subjected to water deficit that received H_2_O_2_ had a higher RWC, 17% higher than plants that did not receive H_2_O_2_, at 12 DAST, the peak of the deficit (Fig. 3c), and at 14 DAST, right after the return of irrigation (Fig. 3d). Plants that received 2× H_2_O_2_ showed RWC equivalent to plants that were well-watered.

Plants exposed to a water shortage showed a decrease in Fv/Fm; nevertheless, foliar H_2_O_2_ treatment promoted a photoprotection mechanism.

**Figure 4:**
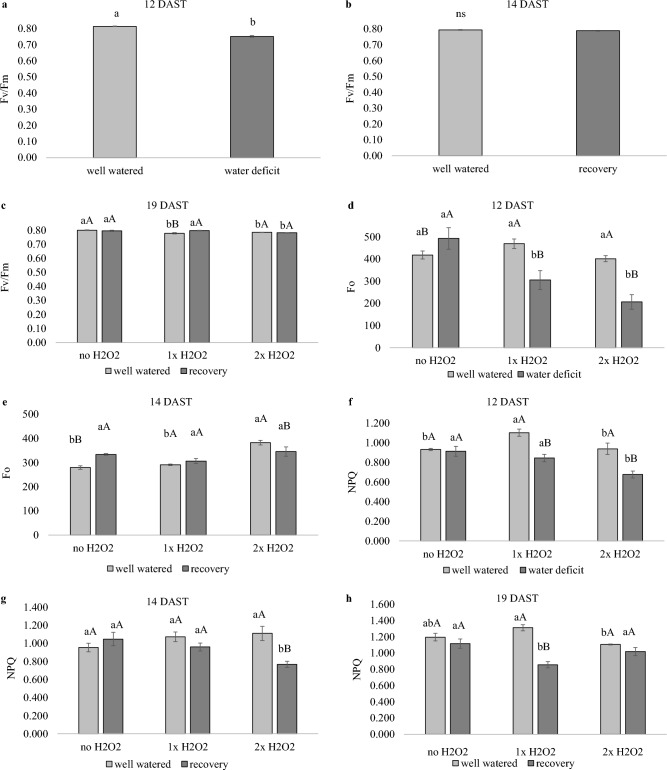
Maximum quantum efficiency of photosystem II (Fv/Fm), minimum fluorescence in the dark-adapted state (Fo) and nonphotochemical quenching (NPQ) of tomato plants well-watered or subjected to water deficit (12 days after the treatments beginning – DAST)/recovered (14 and 19 DAST), and zero, one or two foliar applications of H_2_O_2_. Values correspond to the mean ± standard error (n = 4). Means with equal letters do not differ from each other by the Tukey test (< 0.05), uppercase between water conditions and lowercase between foliar application of H_2_O_2_.

Water-stressed plants had a reduced maximum quantum efficiency of photosystem II adapted to the dark (Fv/Fm) 12 days after beginning treatments (DAST), with Fv/Fm of around 0.75 compared to 0.80 in hydrated plants (Fig. 4a). At 14 DAST, recovered plants did not vary from well-watered plants, and at 19 DAST, plants that received H_2_O_2_ exhibited a modest drop in Fv/Fm (Fig. 4b,c).

At 12 DAST, plants lacking H_2_O_2_ that were exposed to water deprivation had higher minimum dark-adapted fluorescence (Fo) than well-watered plants (Fig. 4d). During the same period, plants subjected to water shortage and given H_2_O_2_ had lower Fo than the others. Plants without recovered H_2_O_2_ remained to have greater Fo at 14 DAST than well-watered plants without H_2_O_2_ (Fig. 4e).

At 12 DAST, plants that got 1× H_2_O_2_ had greater NPQ than well-watered plants, but plants that received 2× H_2_O_2_ had the lowest NPQ among water-stressed plants (Fig. 4g). After watering was restarted, recovered 2× H_2_O_2_ plants continued to have reduced NPQ (Fig. 4h), while recovered 1× H_2_O_2_ plants had lower NPQ (Fig. 4i) at 19 DAST.

### Photorespiration and daily respiration were altered by H_2_O_2_ foliar spray regardless of water conditions

**Figure 5:**
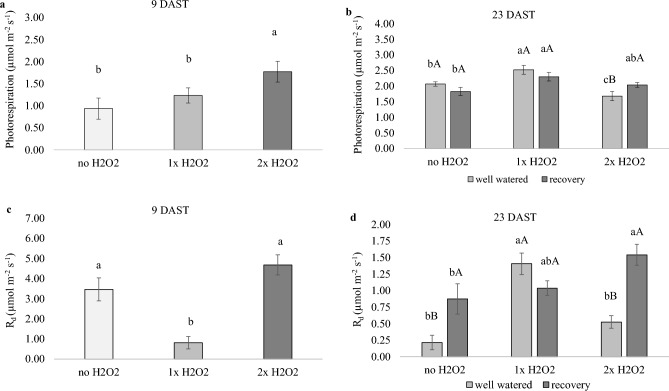
Photorespiration rate and daily respiration (*R*_*d*_) of tomato plants well-watered or subjected to water deficit (9 days after the treatments beginning – DAST)/recovered (23 DAST) and zero, one or two foliar applications of H_2_O_2_. Values correspond to the mean ± standard error (n = 4). Means with equal letters do not differ from each other by the Tukey test (< 0.05), uppercase between water conditions and lowercase between foliar application of H_2_O_2_.

At 9 DAST, plants that got 2× H_2_O_2_ exhibited 89% larger photorespiration than plants that did not get H_2_O_2_ application (Fig. 5a), although well-watered or recovered plants that received 1× H_2_O_2_ showed stronger photorespiration (Fig. 5b). At 9 DAST, daily respiration (*R*_*d*_) was four times lower in plants that got 1× H_2_O_2_ (Fig. 5c) than in plants that did not get H_2_O_2_ treatment. At 23 DAST, well-watered plants treated with 1× H_2_O_2_ had a higher *R*_*d*_ than recovered plants treated with 2× H_2_O_2_ (Fig. 5d).

### H_2_O_2_ foliar spray raised foliar concentrations of reducing sugars and sucrose in water-stressed plants

**Figure 6:**
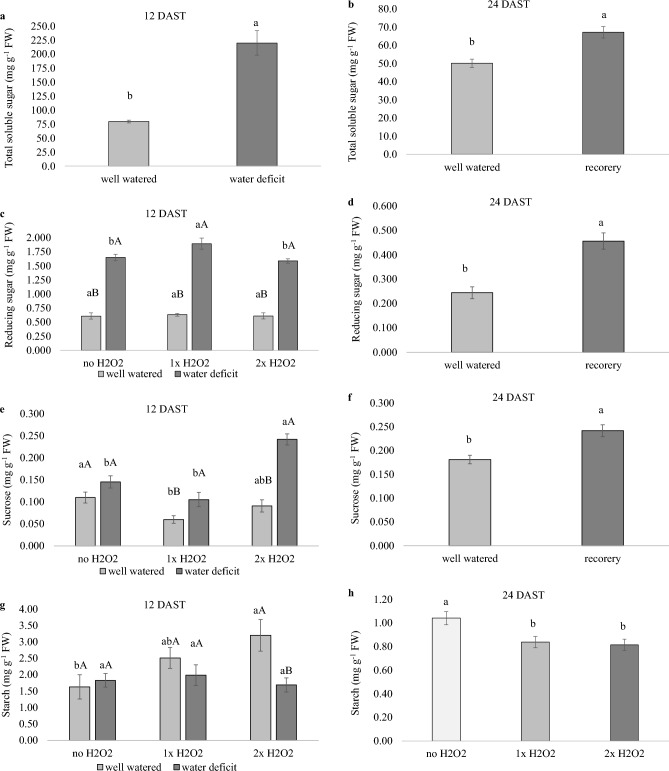
Carbohydrates of tomato plants well-watered or subjected to water deficit (12 days after the treatments beginning – DAST)/recovered (24 DAST) and zero, one or two foliar applications of H_2_O_2_. Values correspond to the mean ± standard error (n = 4). Means with equal letters do not differ from each other by the Tukey test (< 0.05), uppercase between water conditions and lowercase between foliar application of H_2_O_2_.

At 12 DAST, plants exposed to water shortage had greater foliar concentrations of total soluble sugars, reducing sugars, and sucrose than irrigated plants, with the exception of sucrose in plants not exposed to H_2_O_2_ (Fig. 6a,c,e). During the same period, 2× H_2_O_2_ enhanced starch concentration in well-watered plants and sucrose concentration in water-stressed plants (Fig. 6g). At 24 DAST, recovered plants continued to have larger concentrations of total soluble sugars, reducers, and sucrose than well-watered plants (Fig. 6b,d,f), although H_2_O_2_ supply lowered plant starch content (Fig. 6h).

### H_2_O_2_ foliar spray boosted chlorophyll a and accessory pigment concentrations in tomato plants regardless of water supply

**Figure 7:**
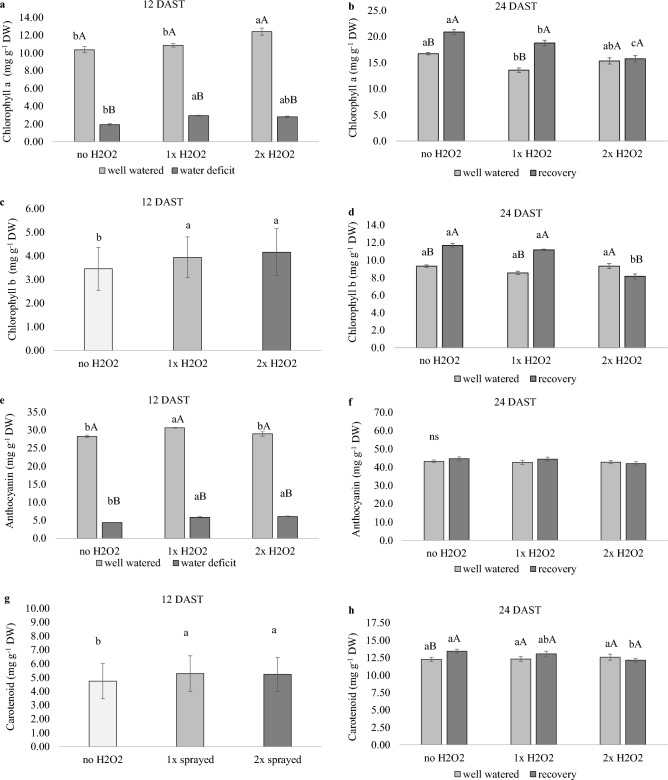
Pigments of tomato plants well-watered or subjected to water deficit (12 days after the treatments beginning–DAST)/recovered (24 DAST) and zero, one or two foliar applications of H_2_O_2_. Values correspond to the mean ± standard error (n = 4). Means with equal letters do not differ from each other by the Tukey test (< 0.05), uppercase between water conditions and lowercase between foliar application of H_2_O_2_.

At 12 DAST, well-watered plants that got 2× H_2_O_2_ had greater chlorophyll *a* concentration than water-stressed plants that received 1× H_2_O_2_ (Fig. 7a). During this time, the application of H_2_O_2_ enhanced the concentrations of the accessory pigments chlorophyll *b*, anthocyanin, and carotenoids (Fig. 7c,e,g). At 23 DAST, recovered plants had greater chlorophyll* a* and *b* concentrations than well-watered plants, except when fed with 2× H2O2 (Fig. 7b,d).

The heatmap revealed that 2× H_2_O_2_ foliar spray significantly altered metabolic reactions, and once watering was resumed, plants that had received 2× H_2_O_2_ displayed responses that were more comparable to well-watered plants that had not received H_2_O_2_.

**Figure 8:**
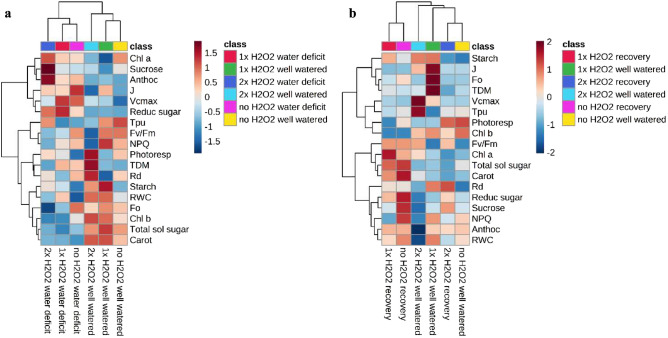
Heatmap and hierarchical cluster analysis for evaluations of maximum photosystem II quantum fluorescence (Fv/Fm), minimum dark-adapted fluorescence (Fo), nonphotochemical quenching (NPQ), maximum RuBisCo carboxylation rate (*V*_*cmax*_), RubP protection rate linked to electron transport (J), RubP defense rate linked to the use of triose phosphate (*TPU*), photorespiration rate (Photoresp), daily respiration (*R*_*d*_), total dry mass (TDM), leaf relative water content (RWC), chlorophyll *a* (Chl a), chlorophyll *b* (Chl b), anthocyanin (Anthoc), carotenoids (Carot), total soluble sugars (Total sol sugar), reducing sugars (Reduc sugar), sucrose (Sucrose) and starch (Starch) in tomato plants well-watered or kept to water deficit/recovered, and zero, one or two foliar applications of H_2_O_2_. (**a**) Evaluations during water deficit and (**b**) after recovery by irrigation return. The heatmap was generated by MetaboAnalyst v5.0 software (https://www.metaboanalyst.ca/).

During the water shortage phase, the heatmap produced two separate primary clusters of well-watered plants and plants in water deficit (Fig. 8a). There was a subdivision of the cluster between plants of the two water conditions, showing less similarity in metabolic reactions for plants that received 2× H_2_O_2_. H_2_O_2_ application impact among plants subjected to water deficiency for Fo decrease and a rise in reducing sugars content stands out. There was a beneficial impact on *J* and total dry mass accumulation among well-watered plants under 1× H_2_O_2_ and 2× H_2_O_2_, respectively.

Two major clusters emerged throughout the time of recovered plants (Fig. 8b). The first was further separated by grouping recovered plants with or without H_2_O_2_. The second cluster was divided among recovered plants that got 2× H_2_O_2_ and well-watered plants that did not get H_2_O_2_, with less similarity in the responses for well-watered plants that received 1× H_2_O_2_ and 2× H_2_O_2_ application. In general, H_2_O_2_-treated well-watered plants had a positive trend for *V*_*cmax*_, *J*, and starch and a negative tendency for total soluble sugars, reducing sugars, and sucrose. Plants that were well-watered but did not get H_2_O_2_ and recovered plants that got 2× H_2_O_2_ exhibited a favorable trend for photorespiration but a negative tendency for starch and total soluble sugars. Meanwhile, plants recovered without H_2_O_2,_ and plants recovered with 1× H_2_O_2_ showed a favorable trend for total soluble sugars, reducing sugars, and carotenoids.

## Discussion

This study found that applying H_2_O_2_ foliar to tomato plants can enhance metabolic acclimation in water-stressed plants and boost RuBisCO carboxylation rate and growth in plants that are always well-watered. Other studies have found that foliar H_2_O_2_ promotes tolerance to abiotic stresses^16,17,20^ and stimulates development in plants grown under appropriate water regimes^18,21^.

Sequential application of H_2_O_2_ (2× H_2_O_2_) provided signaling for metabolic responses that induced tolerance, as evidenced by less water loss from tissues and an increase in photorespiration, which may have contributed to the removal of excess energy in photosystems, important conditions for recovering photoinhibition at the peak of the deficit. Plants that received 2× H_2_O_2_ displayed metabolic reactions like those of well-watered plants that did not receive H_2_O_2_ once watering was restarted (Fig. 8b).

H_2_O_2_ foliar treatment induced alterations in tomato photosynthetic metabolism independent of water status, but it only enhanced growth in well-watered plants. H_2_O_2_ foliar application enhanced *V*_*cmax*_ in tomato plants (Fig. 1a,b), which was also confirmed in other species, suggesting that H_2_O_2_ has the potential to change the Rubisco activation state^22^. In a more recent investigation, Jamaludin et al.^18^ demonstrated that H_2_O_2_ foliar application boosted the expression of the plastid DNA origin rbcL gene, which relates to the RuBisCO component, in *F. deltoideae*, hence increasing photosynthesis. According to the researchers, H_2_O_2_ affects gene expression via protein oxidation, kinase activation, and transduction cascade modulation.

H_2_O_2_ foliar application increased *J* and *TPU* in plants subjected to water deficiency, variables that, in the second evaluation, had a good influence on the dynamics of the photosynthetic process, resulting to better growth in well-watered plants. The key limiting event for photosynthesis in plants with moderate water shortage is stomatal closure, which limits the CO_2_ concentration available for the process^23^. Assimilation reduces as CO_2_ concentration drops, causing a decrease in sucrose phosphate synthase enzyme activity^24^, which may be associated to a decrease in *TPU*.

According to Sharkey^25^, limiting *TPU* capacity by removing the reproductive component causes sucrose buildup and negative feedback for photosynthesis. The water deficiency condition and the necessity for osmotic adjustment are likely to restrict the strength of drainage, resulting in a loss in growth capacity. Higher *TPU* may be connected to increased foliar accumulation of reducing sugars and sucrose (Fig. 6c,d,e,f), which may contribute to tissue hydration, as evidenced by higher RWC of plants subjected to water deficiency and treated with H_2_O_2_ (Fig. 3c,d,e). Higher *TPU* correlates with higher sucrose phosphate synthase enzyme activity, as demonstrated by Ozaki et al.^26^ after feeding H_2_O_2_ to *Cucumis melo* plants.

Only well-watered plants that received 1× H_2_O_2_ showed higher *J* at 23 DAST, which may imply increase activity of additional Calvin-Benson cycle enzymes, in addition to RuBisCO, and production of phosphate trioses with fast RuBP substrate regeneration^27^.* J* can be limited owing to a decrease in the efficiency of the biochemical phase of photosynthesis, or when the light intensity exceeds the capacity for photosynthesis, resulting in increased photorespiration and increased ROS formation as H_2_O_2_.

In isolated chloroplasts, Charles and Halliwell^28^ demonstrated that a high H_2_O_2_ concentration can change the redox state of fructose-1,6-bisphosphatase (FBPase), an important enzyme in the Calvin-Benson cycle, oxidizing it and rendering it inactive, reducing the rate of triose phosphate formation and RuBP regeneration. Due to interference from the second application, which enhanced photorespiration at 12 DAST, the supply of 2× H_2_O_2_ may have lowered FBPase activity, resulting in more endogenous H_2_O_2_ generation and restricted *J* (Fig. 1d).

H_2_O_2_ foliar treatment in well-watered plants resulted in quicker growth and increased mass accumulation, which was more obvious in the well-watered plants that received 1× H_2_O_2_, as the net absorption rate dropped more pronouncedly after the sixteenth DAST (Fig. 2a). Other investigations found comparable findings in various plant species where the H_2_O_2_ supply promoted growth^18,29^. In this investigation, there was a larger dry mass buildup in watered plants that received H_2_O_2_ (Figs. 2g and 3a), which might be attributed to the signaling effect on photosynthetic enzymes, particularly RuBisCO, as revealed by Jamaludin et al.^18^.

Plants subjected to water deficiency and treated with 2× H_2_O_2_ foliar treatment had nearly constant NAR and RGR, indicating sluggish development (Fig. 2a,b). High NAR and RGR are predicted in the early stages of growth for the synthesis of leaf area, but as the cycle progresses, with self-shading, the development of non-photosynthetic tissues, and fruiting, these indices decrease, suggesting a change in the source-sink ratio^30^. Thus, the application of 2× H_2_O_2_ to plants during a period of water deficit altered the growth dynamics as well as the preferential order for investment of photoassimilates, which in this case was more retained in the leaves, as confirmed by LAR, LMR, and SLW (Fig. 2c,d,e) and leaf sucrose concentration (Fig. 6e).

The water shortage reduced the Fv/Fm of these plants in comparison to the well-watered plants (Fig. 4a). Plants under drought stress exhibit a drop in Fv/Fm owing to photosystem damage, surplus energy that cannot be utilized in the biochemical phase of photosynthesis, lower CO_2_ availability due to stomatal closure, and an increase in photorespiration, according to Cao et al.^31^. This situation results in the production of ROS and the peroxidation of membranes critical to the photosynthetic process. There was no variation in Fv/Fm two days after the return of watering, indicating full recovery of the plants, and in the final evaluation, at 19 DAST, the lower Fv/Fm of the well-watered plants that received H_2_O_2_ may be due to self-shading^32^ due to rapid growth and greater mass accumulation (Fig. 2b,c).

Among the plants that did not receive H_2_O_2_, those that were subjected to water shortage exhibited an increase in Fo compared to those that were well-watered (Fig. 4d). Cao et al.^31^ discovered a reduced Fo in the early days of a water shortage, but as the stress period progressed, Fo climbed and stayed greater than the watered control until the conclusion of the stress. Drought-stressed plants, according to Cao et al.^31^ and Demmig et al.^33^, frequently exhibit an increase in Fo because of photosystem degradation produced by ROS, with irreversible dissociation of chlorophyll from the PSII light-harvesting protein complex and reversible inactivation of the PSII reaction center.

Drought-affected plants frequently exhibit greater NPQ, which was not found in this investigation, most likely due to energy being directed to different photoprotection mechanisms (Fig. 4g). NPQ is a photoprotective mechanism that causes excess energy to be dissipated in the form of heat, lowering the potential energy for reactive species formation. The photoprotective mechanism involves the xanthophyll cycle converting violaxanthin to zeaxanthin, a carotenoid crucial in heat dissipation^34^. In this work, we found that well-watered plants exposed to 1× H_2_O_2_ had increased NPQ (Fig. 4g) and carotenoids concentrations (Fig. 7g).

Photorespiration is another key resource for photoprotection of plants under water stress, and 2× H_2_O_2_ treatment boosted its usage (Fig. 5a). Photorespiration is vital for C3 plants, according to Lima Neto et al.^35^ since it uses the dual carboxylase/oxygenase activity of RuBisCO to maintain photosystem integrity under lighted situations with low CO_2_ availability. In a sequence of processes that involve chloroplast, peroxisome, and mitochondria and release CO_2_ and NH_3_, the oxygenase activity of RuBisCO produces one molecule of 3 phosphoglycerate and one molecule of 2 phosphoglycolate. The energy necessary to convert 2-phosphoglycolate into 3-phosphoglycerate for RubP regeneration in these plants may explain the growth halt, since photosynthetic resources may have been diverted to overcoming stress.

H_2_O_2_ supplying also modified *R*_*d*_. During a water deficit, a decrease in *R*_*d*_ in plants treated with 1× H_2_O_2_ (Fig. 5c) may have helped preserve tissue hydration, allowing for more soluble carbohydrate storage, whereas in well-watered plants, reduced use of reserves resulted in greater growth. Higher *R*_*d*_ in well-watered plants that received 1× H_2_O_2_ (Fig. 5d) may imply a greater demand for ATP for sucrose synthesis, as shown by higher *TPU* in the second assessment. During the day, the metabolism of lighted plants must coordinate and satisfy the energy demands of the cells; hence, chloroplasts and mitochondria function in tandem with environmental changes. Thus, photosynthesis produces organic chemicals that are oxidized by respiration to form ATP and reducing agents, which offer energy to cells^6^.

Recovered plants that received 2× H_2_O_2_ had greater *R*_*d*_ (Fig. 5d), which may have decreased total soluble sugar and starch accumulation (Figs. 6h and 8b). Furthermore, in poor climatic situations that impede photosynthesis, such as drought, respiration can digest the surplus reducing energy created in photosystems, providing alternate paths for energy supply and plant recovery^36^. Tomato plants were more resistant to salty stress caused by brassinosteroids, which involved H_2_O_2_ and ethylene. Ethylene buildup boosted stress tolerance by increasing the usage of the alternative oxidase pathway^19^.

Plants with a water deficit had greater concentrations of total soluble sugars (Fig. 6a), which is a common plant organism response to cellular dryness and represents osmotic adjustment to preserve water resources in the leaves^37^. In these plants, 1× H_2_O_2_ increased the concentration of reducing sugars, but 2× H_2_O_2_ increased the concentration of sucrose (Fig. 6c,e). Similar results were reported in Ozaki^26^'s investigation, in which H_2_O_2_ supply increased fructose, glucose, and sucrose accumulation in *C. melo*. The authors propose that the increased activity of enzymes involved in sugar production, such as sucrose phosphate synthase, is to blame for this finding. Plants that got H_2_O_2_ treatment had greater *TPU*, as previously noted in this investigation, correlating with the reported findings. The greater sucrose content (Figs. 6e and 8a) in plants under water deficiency that received 2× H_2_O_2_ was also a result of the decreased growth rate of these plants, with reduced drain strength and carbohydrate buildup in the leaves.

The necessity for osmotic adjustment in plants under water stress involves changes in carbon allocation, with a reduction in starch storage for conversion into soluble molecules with reduced molecular weight^37^. Sugars like glucose and fructose can be employed as an energy source for cellular functions, a building block for the manufacture of specialized and osmoprotective chemicals, and a signaling molecule for the activation of plant defense genes^37,38^. Plants that got 1× H_2_O_2_ and had a greater concentration of reducing sugars may have employed the above-mentioned defensive activities (Fig. 6c).

Supplying 1× H_2_O_2_ to well-watered plants lowered sucrose content at 12 DAST, which agrees with the greatest NAR confirmed (Figs. 6e and 2a). Sucrose export from source tissues to sinks with strong metabolic activity maintains low foliar carbohydrate concentrations, which can generate negative feedback for photosynthesis if held in larger concentrations^39^.

However, in the well-watered plants that received 2× H_2_O_2_, a rise in leaf starch content was seen (Fig. 6g), a finding like that observed in the research by Ozaki et al.^26^ in melon leaves when H_2_O_2_ was supplied. The authors propose that H_2_O_2_ plays an important role in regulating the Calvin-Benson cycle and sugar metabolism, particularly via modulating the activity of enzymes like FBPase and sucrose phosphate synthase. As previously noted, high H_2_O_2_ concentrations may lower FBPase enzyme activity, and this change may encourage starch buildup in the leaves.

At 24 DAST, recovered plants had larger concentrations of total soluble sugars, reducing sugars, and sucrose (Fig. 6b,d,f), perhaps due to slower growth and metabolic adaptations still required for full recovery from the water shortage phase. The drop in starch content was only observed in plants that received H_2_O_2_ (Fig. 6h), which may imply that these plants benefited from the greater concentration of these soluble sugars, resulting in enhanced water resource use and growth signaling.

The water deficit reduced the concentration of leaf pigments in tomato plants due to an increase in ROS concentration, which causes oxidative damage, and an increase in ethylene production, which activates chylase and causes chlorophyll molecules degradation^3,4^, as confirmed in our study (Fig. 7a). In addition to the water deficit, H_2_O_2_ foliar application altered the foliar pigment concentration, with increased chlorophyll *a* concentration found in well-watered plants that got 2× H_2_O_2_, and in water-deficient plants that received 1× H_2_O_2_ (Fig. 7a). Other investigations have found higher chlorophyll *a* and *b* concentrations in plants treated with H_2_O_2_. Habib et al.^40^ discovered that H_2_O_2_ applied to wheat subjected to water deficit increased chlorophyll *a* and total, and attributed this result to increased antioxidant enzyme activity, whereas Jamaludin et al.^18^ discovered an increase in chlorophyll *a* and *b*, as well as carotenoids, in *F. deltoidea* with weekly application of H_2_O_2_. According to Nurnaeimah et al.^41^, H_2_O_2_ protects leaf cells against senescence, but at low concentrations it accelerates chlorophyll breakdown owing to activation of an H_2_O_2_ peroxidase. Depending on the water condition, foliar application of 1 × or 2× H_2_O_2_ may result in chloroplast ultrastructure protection, allowing for a larger concentration of photosynthetic pigments.

The rise in chlorophyll *b*, anthocyanins, and carotenoids (Fig. 7c,e,g) helps to explain the greater concentration of chlorophyll* a* observed, since they are important in dissipating excess energy and maintaining photosystem integrity. Anthocyanin is a plant defense pigment that has antioxidant capabilities against ROS as well as light filtering characteristics that decrease the amount of energy that reaches the photosystem^42^. Anthocyanin accumulation was also linked to the activation of protective functions in tobacco plants under water stress, with changes in carbohydrate metabolism resulting in higher RWC^42^, a result like that observed in this study in plants under water stress that received H_2_O_2_ application (Fig. 7e).

The increase in carotenoids found in plants exposed to H_2_O_2_ may imply a metabolic response with benefits in photosystem protection, resulting in increased development of well-watered plants. Carotenoids regulate surplus energy predominantly by conformational changes in the PsbS protein, which is found in the lumen of the thylakoid and gets protonated when the pH of the medium decreases. Simultaneously, the enzyme deepoxidase activates, resulting in the reduction of violaxanthin to zeaxanthin, which binds to PsbS. This binding causes carotenoid interaction with the light harvest center (LHC), and energy is channeled from chlorophyll to zeaxanthin, which is effective in heat radiation dissipation^5^.

At 24 DAST, recovered plants that did not receive H_2_O_2_ or 1× H_2_O_2_ had greater leaf pigment concentrations than well-watered plants (Fig. 7b,d,h). As previously stated, H_2_O_2_ is essential for chloroplast preservation, and its availability may allow for greater foliar pigment concentrations. We believe that the water shortage had the capacity to initiate H_2_O_2_ signaling after recovery, and that the administration of 1× H_2_O_2_ was sufficient to keep this signal going. However, the addition of 2× H_2_O_2_ appears to have started a process to eliminate this ROS, resulting in chlorophyll *a* and carotenoids concentrations comparable to well-watered plants (Fig. 7b,h). Another theory is that recovered plants that had 2× H_2_O_2_ treatment collected more ethylene, with chlorophyll degradation^3^ promoting a higher *R*_*d*_, which may be associated to the usage of alternate pathways triggered by ethylene accumulation^19^.

In this study, we found that foliar application of 1 mM H_2_O_2_ helped tomato photosynthetic adjustment by increasing the maximum rate of RuBisCO carboxylation by 69% in continually well-watered plants. H_2_O_2_ treatment resulted in an increase in dry mass of up to 37% in these plants, indicating that it acts as a growth stimulant. 2× H_2_O_2_ increased stress tolerance in plants subjected to water shortage, with a reduction of just 18% in the maximal rate of RuBisCO carboxylation, but in plants that did not receive H_2_O_2_ treatment, the reduction was 86% in comparison to well-watered plants. Plants exposed to a water shortage and given 2× H_2_O_2_ stored sucrose in the leaves and had a 17% higher RWC than plants not given H_2_O_2_. Thus, H_2_O_2_ foliar treatment can be used in tomato management to induce drought tolerance or to boost photosynthetic activity and dry mass formation in well-watered plants.

## Material and methods

### Experimental conditions

The experiment was carried out in a Van der Hoeven pad fun greenhouse at 25 ± 5 °C between October and December 2020. The greenhouse and all evaluations were conducted in the UNESP Biosciences Institute's Biodiversity and Biostatistics Department, Campus Botucatu, São Paulo, Brazil (22°49′10″ S, 48°24′35″ W, and an average height of 800 m). During the experiment, the following environmental parameters were verified: a photosynthetic photon flux density of 836 ± 200 µmol m^2^ s^−1^, a relative air humidity of 43 ± 10%, and an ambient CO_2_ concentration of 410 ± 5 µmol mol^−1^.

### Plant material

Tomato plants (*S. lycopersicum* L. cv. Micro-Tom subtype wild) were utilized as a model, and all procedures employed in this study were in conformity with applicable rules and laws. The seeds germinated on vermiculite-filled trays. Plants were selected and transplanted into separate 1 dm^3^ pots with 130 g of vermiculite as substrate 12 days following seedling emergence. All plants were fertilized every two days with nutritional solution n°2^43^, at first with 25% ionic force and then with 50% after seven days. Due to blooming (45 days following seeding), all treatments were allocated on November 19th.

### Experimental trial design, treatment application, water deficiency establishment and recovery after watering return

Tomato plants with exogenous H_2_O_2_ delivery were tested in two ways: without and with a time of water deprivation. The experiment consisted of two simultaneous tests with four repeats in a 2 × 3 factorial trial design. The first test included plants that were continually well-watered and plants that were water-stressed and received foliar water treatments (without H_2_O_2_) or one or two H_2_O_2_ foliar sprays (1× H_2_O_2_ and 2× H_2_O_2_, respectively). The second test used the same settings as the first, but plants with a lack of water were recovered due to watering return (well-watered plants and recovered plants, without H_2_O_2_, 1× H_2_O_2_ or 2× H_2_O_2_).

H_2_O_2_ application treatments kicked off the trial. Plants without H_2_O_2_ were pulverized with a solution (deionized water + nonionic adjuvant), plants 1× H_2_O_2_ were pulverized with the same solution and + 1 mM H_2_O_2_, and plants 2× H_2_O_2_ were pulverized twice (24 h between treatments). The hydrogen peroxide utilized was BAKER™ 30% stabilized hydrogen peroxide. All plants were pulverized twice, once for each treatment. A CO_2_ pressured costal sprayer with a complete conical tip and a pressure of 0.3 kgf per 31 cm^2^ was utilized, with 8 mL of solution applied to each plant.

During all assessment periods, well-watered plants got water every two days after foliar sprays. When a CO_2_ assimilation rate less than 0 µmol m^−2^ s^−1^, measured with IRGA, was verified in plants that did not receive H_2_O_2_, the water supply was conducted until percolation and substrate natural drainage, whereas plants with water deficit remained without hydric repositioning for 12 days. Watering with nutritive solution at 50% was employed in all treatments at the end of the twelfth day after the starting of all treatments (DAST). All plants were watered every two days till the experiment finished on the 26th DAST.

### Chlorophyll fluorescence and RuBisCO carboxylation curve

Infrared gas analyzer (IRGA) equipment, model GSF 3000, Walz, and a portable modulated light fluorometer (LED-ARRAY/PAM-Module 3055-FL) attached to the GSF 3000 were used to measure chlorophyll *a* fluorescence. After 30 min in the dark, leaves were exposed to an actinic light pulse of 4500 μmol m^−2^ s^−1^ to get Fm (maximum dark-adapted fluorescence). Maximum photosystem II quantum efficiency (Fv/Fm), lowest fluorescence in the dark-adapted condition (Fo), and nonphotochemical quenching (NPQ) were also assessed. Measurements were taken on the second or third completely grown leaf between 9 a.m. and 11 a.m. The evaluations were taken out on the 12th (water deficit peak), 14th (48 h after watering return) and 19th DAST.

According to Sharkey et al. (2007)^27^, the photosynthetic response curve to CO_2_ concentration was performed (*A/Ci* curve, where *Ci* corresponds to the concentration of CO_2_ in the substomatal chamber, expressed in µmol mol^−1^) to analyze the effects of treatments on diffusion and biochemical limitations for CO_2_ assimilation. The experiments began in the gas exchange chamber with a CO_2_ concentration of 400 µmol mol^−1^ and were gradually lowered to 300, 200, 100, 50, and 0 µmol mol^-1^. The concentration was subsequently increased to 400, then 700, 1000, 1300, 1600, and finally 2000 µmol mol^−1^ CO_2_. The measurements were taken with a 1200 μmol m^-2^ s^-1^ irradiance and a 750 μmol m^−2^ s^−1^ airflow.

With the “*A/Ci* curve fitting utility version 2007.1” software^27^, variables such as RuBisCO carboxylation maximum velocity (*V*_*cmax*_—µmol m^-2^ s^-1^), RubP regeneration rate linked to electron transport (*J*—µmol m^−2^ s^−1^), RubP regeneration rate linked to phosphate trioses usage (*TPU*—µmol m^−2^ s^−1^) and daily respiration rate (*R*_*d*_—µmol m^−2^ s^−1^) were estimated. According to Sharkey et al.^44^, the photorespiration rate (µmol m^−2^ s^−1^) was also determined using RuBisCO kinetics. The assessments were carried out on 9 and 23 DAST.

### Growth analysis

Leaf area measurement (LA) using a LI-3100C Area Meter was utilized for growth analysis, and leaf dry biomass (LDM) assessment and total dry biomass (TDM) were employed after material drying in an air forced circulation stove at 40 °C until constant dry mass was reached. The sample took place at 0, 7, 14, 21, and 35 DAST.

According to Benincasa (2003)^45^, the growth index was derived using the leaf area ratio (LAR), specific leaf area (SLA), specific leaf weight (SLW), leaf mass ratio (LMR), net assimilatory rate (NAR), and relative growth ratio (RGR).

$$ {\text{LAR }}\left( {{\text{dm}}\,{\text{g}}^{ - 1} } \right) \, = {\text{ LA}}/{\text{TDM}} $$

$$ {\text{SLA }}\left( {{\text{dm}}{\text{ g}}^{ - 1} } \right) \, = {\text{ LA}}/{\text{LDM}} $$

$$ {\text{SLW}}\left( {{\text{dm}}\;{\text{g}}^{ - 1} } \right) \, = \, 1/{\text{SLA}} $$

$$ {\text{LMR }} = {\text{ LDM}}/{\text{TDM}} $$

$$ {\text{NAR}}\left( {{\text{g}}\,{\text{dm}}^{ - 2} {\text{day}}^{ - 1} } \right) = \left( {P2 - P1/{\text{t}}2{-}{\text{t}}1} \right) \, \times \, \left( {{\text{In LA}}2{-}{\text{In LA}}1} \right)/\left( {{\text{LA}}2{-}{\text{LA1}}} \right) $$

$$ {\text{RGR }}\left( {{\text{g}}\;{\text{g}}^{ - 1} \;{\text{day}}^{ - 1} } \right) \, = \, \left( {\ln \,P2{-}\ln \,P1} \right)/\left( {t2{-}t1} \right) $$

where *P*1 and *P*2 are two successive samples and t is the elapsed time.

### Relative water content in tomato leaves

Five foliar discs (1 cm^2^) and fresh mass determination (FM) were used to determine the relative water content in tomato leaves. Following weighing, all discs were put on Petri plates with filter paper and submerged in deionized water for 24 h at 5 °C before being weighed again to calculate turgid mass (TM). After 48 h of drying at 60 °C in a stove with forced air circulation, the disc dry mass (DM) was calculated. According to Elsheery and Cao’s technique^46^, RWC was determined using the equation RWC% = (FM—DM)/(TM—DM) × 100. The assessments were carried out at 12, 14, 21, and 24 DAST.

### Carbohydrates

At 12 and 24 DAST, the total soluble sugar concentration, as well as the reducing sugar, sucrose, and starch contents of tomato leaves, were measured. According to Garcia et al.^47^, carbohydrate extraction was conducted using 100 mg of macerated and frozen fresh leaves. Total soluble sugar, reducing sugar, and sucrose were determined using an 80% ethanol solution, followed by 15 min of heating at 80 °C in a water bath and 15 min of centrifugation at 4 °C at 12,000 × g for 15 min. Starch extraction was performed using a 52% perchloric acid solution, agitation in chilled water for 5 min, and centrifugation at 4 °C at 10,000 × g for 5 min. All carbs were measured in mg g^−1^ of fresh materials.

Total sugar was quantified using the reagent antron and standard glucose curves in a spectrophotometer at 620 nm, as described by Morris^48^ and Yemm and Willis^49^. According to Miller^50^, sugar reduction was measured. DNS (3,5 dinitrosalicylic acid + copper hydroxide + sodium potassium tartrate) was employed as the reagent, and a standard glucose curve was produced using absorbance data in a spectrophotometer at 540 nm. Sucrose was measured in accordance with Passos^51^. Antron and potassium hydroxide were employed as reagents, and the absorbance was measured in a spectrophotometer at 620 nm. Morris^48^ and Yemm and Willis^49^ methods were used to quantify starch. The reagent used was antron, a standard glucose curve was also employed, and the absorbance was measured in a spectrophotometer at 620 nm. All readings were carried out in duplicate.

### Foliar pigments

Foliar pigment extraction was evaluated using 50 mg samples of fresh leaves macerated and frozen according to Sims and Gamon^52^, and the following solutions were used: 0.2 M Tris (pH 7.8) in 80% acetone. The samples were homogenized with Tris-acetone solution before being frozen for one hour with vortex agitation. The macerated samples were centrifuged for 5 min at 4 °C at 1000 × g. Supernatant absorbance was measured at 663 nm (chlorophyll *a*), 647 nm (chlorophyll *b*), 537 nm (anthocyanin), and 470 nm (carotenoid). The results were given in mg of pigment g^−1^ dry mass. The evaluations were carried out at 12 and 24 DAST. The pigment calculation equations were as follows:

$$ {\text{Chlorophyll}}\,a = \, 0.01373 \times \left( {A663} \right){-}0.000897 \times \left( {A537} \right){-}0.003046 \times \left( {A647} \right) $$

$$ {\text{Chlorophyll}}\,b = \, 0.02405 \times \left( {A647} \right){-}0.004305 \times \left( {A537} \right){-}0.005507 \times \left( {A663} \right) $$

$$ {\text{Anthocyanin }} = \, 0.08173 \times \left( {A537} \right){-}0.00697 \times \left( {A647} \right){-}0.002228 \times \left( {A663} \right) $$

$$ {\text{Carotenoid}} = (A470{-}\left( {17.1 \times \left( {{\text{Cla }} + {\text{ Clb}}} \right){-}9.479 \times \left( {{\text{anthocyanin}}} \right)} \right)/119.26 $$

### Statistical analysis

Data were subjected to a double factorial variance analysis, and all differences between treatments and their interactions were examined for each assessment period. Tukey's test (5% probability) was used to compare the findings.

MetaboAnalyst 5.0 software was used to generate a heatmap graphic that created cluster connecting treatments and evaluated variables for plants that had a water deficit phase and recovered plants.

## Data Availability

This published paper includes all data produced or analyzed during this project.
